# Microstructures and Properties of NbC-Reinforced Ni-Based Coatings Synthesized In Situ by Ultrasonic Vibration-Assisted Laser Cladding

**DOI:** 10.3390/ma16041704

**Published:** 2023-02-17

**Authors:** Guofu Lian, Kun Yue, Jiayi Zeng, Meiyan Feng, Ruqing Lan, Linghua Kong

**Affiliations:** 1 School of Mechanical and Automotive Engineering, Fujian University of Technology, Fuzhou 350118, China; 2 Fujian Key Laboratory of Intelligent Processing Technology and Equipment, Fujian Institute of Engineering, Fuzhou 350118, China

**Keywords:** ultrasonic vibration, laser cladding, microstructure, hardness, friction coefficient

## Abstract

This paper aims to explore the mechanism of an ultrasonic applied field on the microstructures and properties of coatings, and clarify the evolution of the molten pool under different ultrasonic frequencies. The Taguchi experimental design method was adopted in this paper. NbC-reinforced Ni-based coatings were in situ synthesized by laser cladding to investigate the effects of ultrasonic vibration process parameters on the microstructure, pore area, microhardness, and wear resistance of the cladding layer. The results show that the pore area decreases first and then increases as ultrasonic power increases from 600 to 900 W and ultrasonic frequency from 23 to 40 kHz. On the contrary, the hardness and wear resistance increase at first and then decrease. The pore area is minimized at 800 W ultrasonic power and 32 kHz ultrasonic frequency, and the hardness is maximized at 600 W ultrasonic power and 40 kHz ultrasonic frequency. Meanwhile, the highest wear resistance can be obtained when ultrasonic power is 700 W and ultrasonic frequency is 32 kHz. Based on the phase structure analysis, the cladding layer mainly consists of FeNi_3_, NbC, B_4_C, and CrB_2_. Ultrasonic vibration will not change the phase composition of the layer. Combined with the varying G/R value and cooling rate, the reasons for the change in grain morphology in different areas were analyzed to reveal the evolution mechanism of the molten pool under the influence of ultrasound.

## 1. Introduction

Laser cladding is a surface modification technology [1,2] with simple operation, a short cooling time, and small heat-affected zones [3,4,5]. This technology has been widely used in the aerospace, shipbuilding, automobile, and other industries. Laser cladding repair equipment can prepare high-performance alloy surfaces on the metal substrate of parts and significantly improve the surface properties in terms of wear resistance, corrosion resistance, heat resistance, oxidation resistance, and electrical characteristics. Laser cladding can not only extend the service life of new parts but also repair worn parts and restore their original dimensions to reduce the cycle of replacing parts. Laser cladding reinforcements are usually prepared by direct addition or in situ synthesis methods. The in situ synthesis is a method that improves the performance of cladding layers by adding elements to react in the molten pool to generate target reinforcements. The in situ synthesized reinforcements have received extensive attention recently [1,2,6], as they significantly improve the hardness, wear resistance, and corrosion resistance of the substrate [7,8]. However, defects such as pores and cracks are easily formed in coatings due to the rapid cooling and heating of laser cladding [9]. Zhang et al. [10] preset TiC-Ni35 composite powders on the surface of AISI 1045 steel to analyze the effect of different laser energy densities and powder ratios on coating morphologies and properties. The hardness and wear resistance of coatings were improved with the increased laser energy density and the decreased TiC powder ratio. The defect rate of coatings was significantly affected by the laser energy density. Li et al. [11] used laser cladding technology to prepare Fe60 + (0–30 wt%) WC mixed powder coatings. They reported that the coatings had good properties without obvious defects as the added WC was controlled at 20 wt%, but the properties decreased and the porosity defect increased with further increasing the reinforcement phase. Accordingly, cladding defects cannot be completely eliminated by adjusting process parameters. In order to improve the forming quality and properties of the coating, the introduction of external fields such as ultrasonic vibration technology has become a new research hotspot.

Ultrasonic vibration is an auxiliary field that affects the molten pool through ultrasonic waves. The ultrasonic wave interacts with materials in the molten pool in the process of laser cladding to produce cavitation and thermal effects. The effects vary the subcooling degree of the local molten pool, thus refining grains [12,13] and uniforming the structure [14,15,16] to a certain extent. Therefore, ultrasonic vibration can effectively upgrade the internal microstructure of coatings by reducing internal defects such as cracks and pores and improving powder agglomeration [17]. Zhuang et al. [18] noted that the macro-forming quality of coatings can be improved by changing the ultrasonic amplitude. The ultrasonic vibration effect was optimal when the amplitude was 17.5 μm. Xiao et al. [19] prepared iron-based amorphous coatings with and without ultrasonic vibrations. By comparison, the crystalline region at the coating bottom with ultrasonic vibrations increased from 46 to 81 μm. Li et al. [20] introduced ultrasonic vibrations to the laser cladding process and discovered that the introduction of ultrasonic vibrations destroyed dendrites and refined grains in the coating. The surface hardness and wear resistance of coatings decreased as ultrasonic powers were 600, 700, and 900 W, while the hardness and wear resistance were improved at 800 W ultrasonic power. Han et al. [21] established an ultrasonic-assisted laser cladding system based on a disc ultrasonic motor. The 316L powders were clad on the surface of ASTM1 1045 steel. The results indicated that the ultrasonic vibration on the surface can refine the bottom and top microstructures of coatings. Zhang et al. [22] fabricated ceramic particle-reinforced iron-based coatings by ultrasonic vibration-assisted laser cladding. They found that ultrasonic vibrations reduced the profile roughness and increased the dilution of coatings. Most of the above studies used a single-factor method to investigate the impact of ultrasonic power or frequency on the coating morphology, but did not systematically explore the coupling influence of both parameters on the coating morphology and performance.

Currently, ultrasonic vibrations have been applied in laser cladding technology. However, few studies explore the influence mechanism of ultrasonic vibration on the microstructure and properties of in situ synthesized coatings. This paper adopts the Taguchi experimental design method and synthesizes in situ NbC by ultrasonic vibration-assisted laser cladding, aiming to improve the properties of coatings and reduce the defects to reveal the action mechanism of the in situ synthesized metal phase. The research investigates the effects of ultrasonic power and frequencies on the pore area, phase composition, hardness, and wear resistance of coatings. The results provide empirical evidence for the preparation of strengthening coatings under ultrasonic fields.

## 2. Materials and Methods

### 2.1. Materials

**Figure 1:**
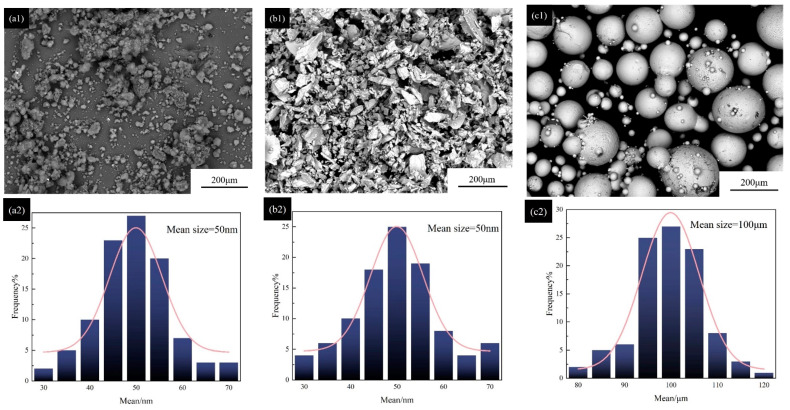
Statistical analysis of powder morphology and particle size: (**a1**,**a2**) B_4_C; (**b1**,**b2**) Nb; (**c1**,**c2**) Ni45.

AISI 1045 steel was selected as the substrate with a size of 40 × 20 × 10 mm in the experiment. B_4_C (99.9% purity), pure Nb powders (99.9% purity), and Ni45 powders were used as laser cladding materials. The particle sizes of B_4_C and Nb powders range from 30 to 70 nm (see Figure 1(a1–b2); the particle size of Ni45 powders varies from 80 to 120 μm (see Figure 1(c1,c2)). Table 1 provides the chemical composition of powders. Based on Figure 1(a1–c2), most B_4_C and Nb powders have particle sizes of approximately 50 nm, and Ni45 powders have a size of approximately 100 μm. B_4_C powders have slight agglomeration. The optimum powder ratio of Nb and B_4_C was obtained through the preliminary experiment. The powder ratio of Nb to B_4_C is at 1:1.3 (atom ratio of Nb and C), and Ni45 powders account for 80% of the total volume of the composed powders.

### 2.2. Ultrasonic Vibration-Assisted Laser Cladding Progress

**Figure 2:**
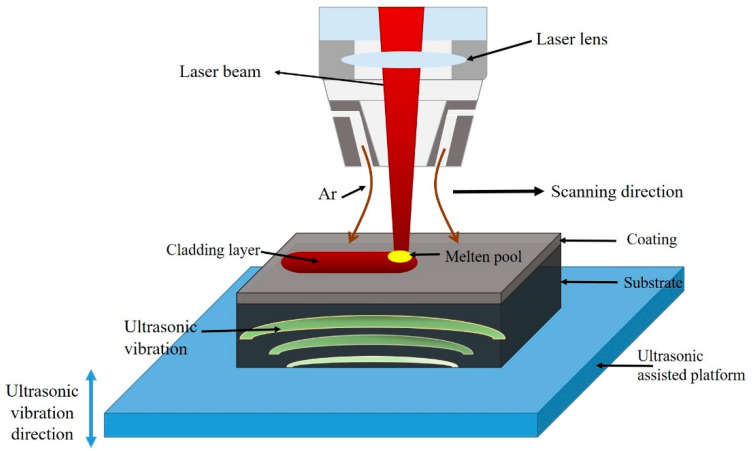
Ultrasonic-assisted laser cladding experimental system.

The substrate surface was cleaned with ethanol before laser cladding. Then, the tablet press was used to preset the tablet under the pressure of 100 MPa, and the powder thickness was fixed at 1 mm. After that, the preset substrate was dried at 120 °C for 3 h in a vacuum dryer. During laser cladding, the output power of the ultrasonic generator ranged from 600 to 900 W, and the frequency from 23 to 40 kHz. To ensure the influence of ultrasonic vibrations on the cladding process, the vibration plate was arranged at the substrate bottom, and the substrate was clamped on the vibration plate, vibrating up and down. Figure 2 presents the schematic of ultrasonic-assisted laser cladding experimental system.

The laser cladding system includes a laser (YLS-3000, IPG, Burbach, Germany), a water cooler (TFLW-4000WDR-01-3385, Sanhetongfei, Hebei, China), a gas-carrying powder feeding system (GZ-DPSF-2, Jiangsu, China), a multi-degree-of-freedom industrial machine arm (M-710iC/50, FANUC, Yamanashi-ken, Japan), a PLC control system (SX14-012PULSE System, China), and a laser cladding head (FDH0273, Lasermech, Novi, MI, USA). Argon was selected as the protective gas in the cladding experiment, and the diameter of the laser spot was 3 mm.

### 2.3. Test Method

A preliminary experiment was conducted to identify the optimum process parameters, including 1700 W laser power, 4 mm/s scanning speed, 8 NL/min gas flow, and 10 mm defocus amount. The optimal powder ratio of Nb and B_4_C was determined to be 1:1.3. The L16_(4×2)_ Taguchi test design was used to investigate the effects of ultrasonic power and frequencies on the pore area, hardness, and wear volume of coatings. Table 2 outlines the experimental process parameters and levels. The experimental results are shown in Table 3.

The cladding samples were corroded in 4% nitric acids for 30 s after wire-electrode cutting, mounting, and polishing. The coating hardness was measured with a microhardness tester (MVA-402TS, HDNS, Shanghai, China) under 1000 gf force and 15 s load holding time. The coating morphology was observed using a scanning electron microscope (TM3030Plus, Hitachi, Chiyoda, Tokyo, Japan). The element composition and distribution were tested by the energy spectrometer (Model 550i, IXRF, Austin, TX, USA). The system parameters of X-ray diffraction analysis (X-Pert Pro MPD, Almelo, The Netherlands) include Cu Kα radiation at 400 kV and 200 mA (λ = 0.15418 nm), 2θ ranging from 10° to 90°, a scanning step at 0.05°, and a measurement time at 10 s per step. Using friction and a wear tester (UMT-2, Bruker, Billerica, MA, USA), the wear resistance was measured in a reciprocating wear testing with the Tungsten steel as friction pair at 30 N loading force. Image-J (1.8.0) software was used to measure cladding area (*CA*) and porosity area (*PA*), and the porosity ratio is calculated by Equation (1).

(1) $$Porosityratio=\frac{PA}{CA}\times 100\%$$

## 3. Results and Discussion

### 3.1. Phase Composition

**Figure 3:**
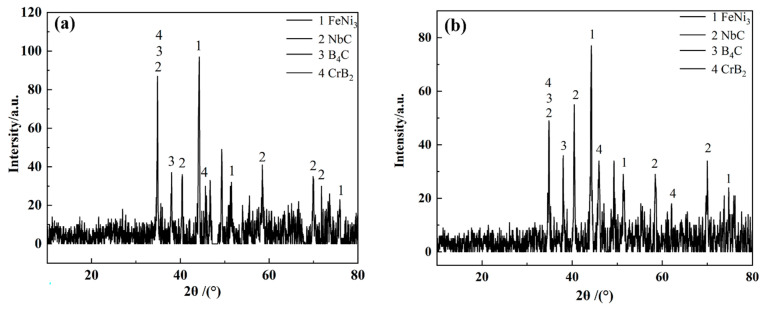
XRD patterns of coatings: (**a**) with and (**b**) without ultrasonic vibrations.

Figure 3 provides the phase compositions of composite coatings with and without ultrasonic vibrations. Based on the figure, the coating phase consists of FeNi_3_, NbC, B_4_C and CrB_2_, indicating a similar phase composition whether ultrasonic vibrations are applied or not. Ultrasonic vibrations do not affect the phase compositions of in situ synthesized NbC and cladding layers.

**Figure 4:**
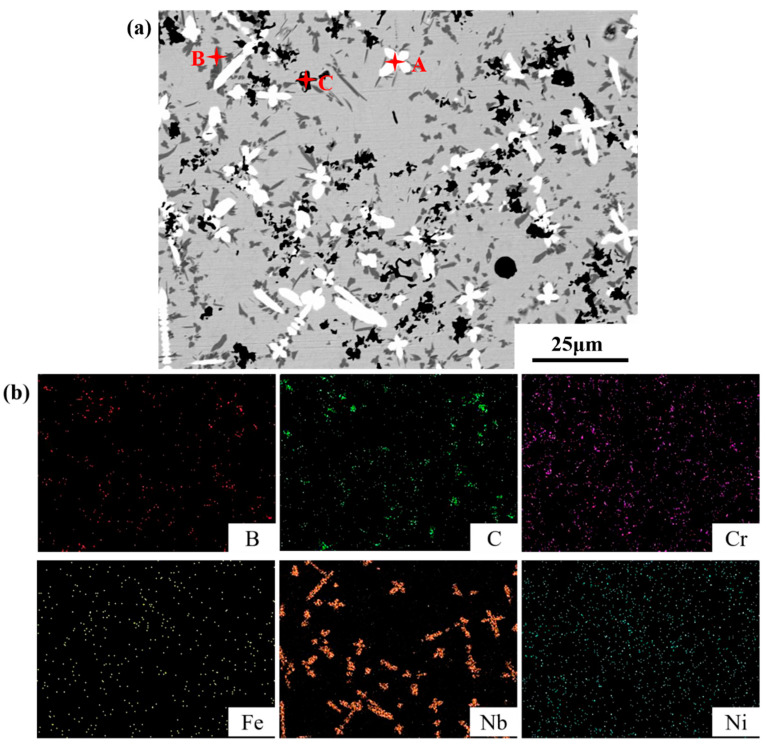
Microstructure (**a**) and element distribution (**b**) of coatings with ultrasonic vibrations.

Coatings were analyzed by an EDS test to identify the phase morphology (see Figure 4 and Table 4). Figure 4a indicates three kinds of color particles—silver flower-like, black flocculent, and gray cubic particles. The content of Nb atom in the silvery white material at point A is 52.03%, and the content of C atom is 36.85%. Combining with the XRD results, it is speculated that it is an in situ synthesized reinforcing phase NbC. The content of Cr element in the gray massive material at point B is up to 40.92%, containing relatively high C and Fe, which is presumed to be the strengthening phase of Cr compound. The black area of point C is rich in C, and the content reaches 54.06%. The C in the coating mainly comes from the decomposition of B_4_C, which is presumed to be the residual C atom of the reaction.

**Figure 5:**
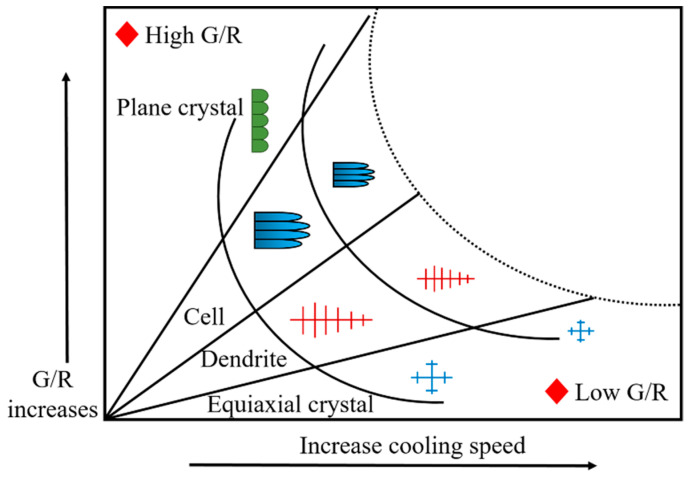
Grain growth at the coating bottom.

Due to the big difference in the grain morphology in coatings, the investigation of the grain-forming factors can reveal the solidification mechanism of the molten pool. Figure 5 demonstrates that during the molten pool solidification, grain morphologies depend on the molten pool temperature gradient (*G*) divided by grain growth rate (*R*). The grain size is determined by the product of *G* and *R*. The morphology of grains changes with the decrease in *G/R* value, and the grain size is reduced as the cooling rate increases.

**Figure 6:**
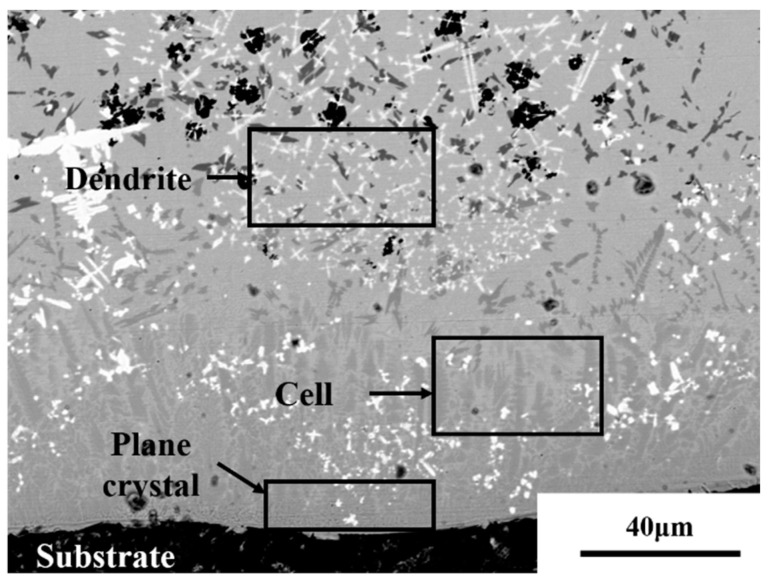
Schematic diagram of grain growth at the bottom of cladding layer.

Figure 6 shows the grain growth at the coating bottom. The heat diffuses rapidly to the substrate with a high *G/R* value at the coating bottom (namely the dilution rate area). The high *G/R* ratio leads to the plane growth of grains at the bottom of coatings, creating plane crystals. The planar crystals nucleate at the fusion line of the substrate and grow toward the internal coating along the opposite direction of the heat diffusion [23]. The growth rate of grains in the middle of the dilution rate region increases with decreasing *G/R* value, leading to grain transformation from planar to cellular crystals. Grains gradually grow into columnar dendrites with a further decreased *G/R* value. The *G/R* value determines the grain size, which gradually decreases as the cooling rate accelerates [24]. It is known that cavitation bubbles generated by ultrasonic vibration will produce instantaneous high temperature and high pressure on the surrounding solution at the moment of collapse, increasing the local temperature gradient (*G*). This gives rise to an increase in *GR* and a decrease in grain size. So, ultrasonic vibration can refine grains [25].

(2) $$\Delta G_{v}=\frac{-L_{m}\Delta T}{T_{m}}$$

where Δ*T* is the subcooling degree; Δ*Gv* is the free-energy difference; *T_m_* is the melting point; and *L_m_* is the latent heat of crystallization. Based on Equation (2), the free-energy difference increases with the increased subcooling degree. The higher free-energy difference increases crystallization driving forces and accelerates crystallization rates. Therefore, the equation suggests a relationship between nucleation rate and subcooling degree.

### 3.2. Influences of Ultrasonic Vibrations on the Pore Area

**Figure 7:**
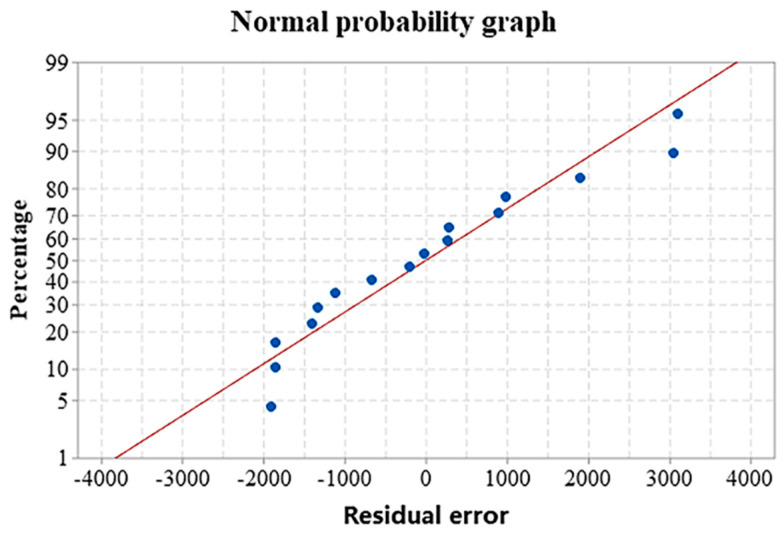
Normal probability of the pore area.

The experimental results were analyzed by a fitting regression model. Figure 7 provides the normal probability of the pore area. The residuals have an S-shaped distribution around the predicted line, indicating a normal distribution of the data. Equation (3) shows the mathematical model of the pore area according to the regression analysis. Table 4 outlines the results of the variance analysis.

(3) PoreArea=368,327−482×UP−10,314×F+0.2371×UP×UP+118.9×F×F+3.421×UP×F

According to Table 5, DF is the amount of information in the data, which is used by analysis to estimate the value of unknown population parameters. The adjusted sum of squares (Adj SS) is a measure of variation in different components of the model. The adjusted mean square (Adj MS) measures the variance of an item or model interpretation. The F value is the test statistic used to determine whether the item is associated with the response. *p* value is a probability used to measure the evidence that negates the original hypothesis.

The *p* value is less than 0.001, indicating a high precision of the model. As the indicators of the model precision, R^2^, R^2^_adj_ and R^2^_pre_ are closer to 100%, suggesting higher fitting accuracy of the model with smaller errors. The high fitting accuracy is also exhibited by the slight difference between R^2^_adj_ and R^2^_pre_ with less than 20%. Based on the significance level analysis, the process parameters are significant when the *p* value is lower than 0.05. Table 5 displays that UP, F, and the interaction items between UP and F have significant effects on the model.

**Figure 8:**
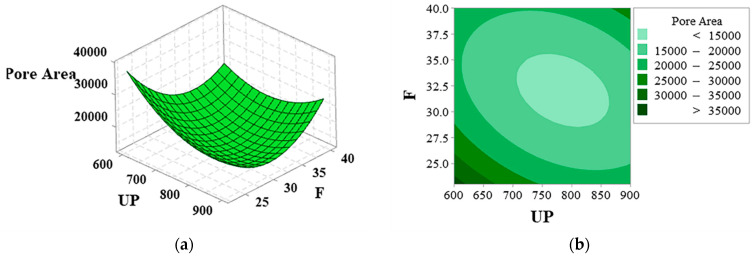
UP and F interaction diagram: (**a**) surface diagram and (**b**) contour diagram.

Figure 8a,b are the surface and contour plots among the pore area, ultrasonic power, and frequency. The pore area decreases and then increases with increased ultrasonic power and frequencies. The pore area is minimized at approximately 800 W ultrasonic power and approximately 32.5 kHz amplitude.

**Figure 9:**
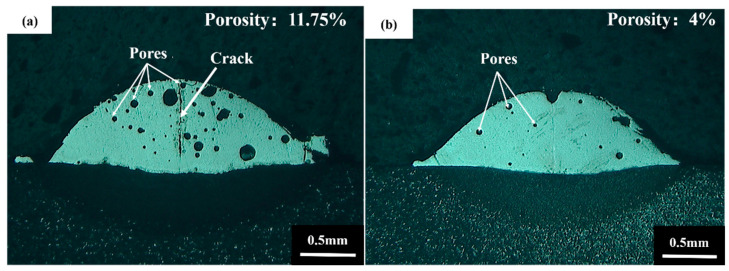
Coating morphologies: (**a**) without ultrasonic vibration; (**b**) with ultrasonic vibration (UP = 800 W, F = 32 kHz).

Figure 9a,b present the coating morphologies with and without ultrasonic vibrations under the same process parameters (LP = 1700 W, SS = 4 mm/s). Figure 9a indicates that coatings without ultrasonic vibrations have cracks and pores with an 11.75% porosity rate. B_4_C is decomposed in the formation of the molten pool to produce C atoms. C is oxidized to generate gas during laser cladding. As the molten pool is solidified, part of the bubbles cannot escape in time due to the rapid solidification speed, leaving pores in the cladding layer. Figure 9b demonstrates that the coatings under ultrasonic vibrations do not have large pores with a 4% porosity rate. Lower porosity suggests that the cavitation effect caused by ultrasonic vibrations can facilitate gas escape in the molten pool, thus eliminating coating defects.

**Figure 10:**
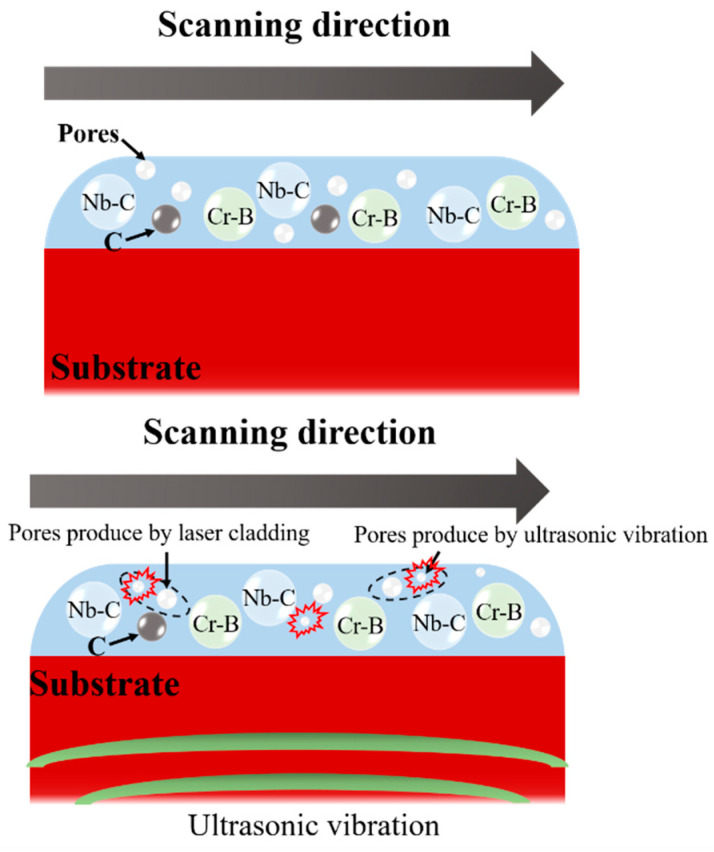
Mechanism of pore elimination [27].

Based on Figure 10, the solution is torn by the local transient negative pressure during the negative pressure phase of ultrasonic vibration. A low-pressure cavitation bubble is formed in the molten pool. When the ultrasonic positive pressure phase is applied, the pressure around the cavitation bubble reaches the threshold of the pool tension. Consequently, the cavitation bubble rapidly shrinks and collapses. At the moment of cavitation bubble collapse, instantaneous high temperature and high pressure will be generated in the surrounding local area. The huge pressure gradient due to high temperature will create micro-jet around the bubble to form local excitation wave and shatter the surrounding bubbles. Therefore, ultrasonic vibration can effectively reduce the pore area of the coating [26].

With the further increase in ultrasonic power, the enhanced cavitation effect will produce large volume of cavitation bubbles in the coating, according to Stokes’ Law (see Equation (4)).

(4) F=6πηvR

where *F* is the friction between fluid and particle; *R* is the radius of the sphere; *v* refers to the velocity of the sphere relative to the liquid; *η* is the viscosity coefficient of the liquid. The increase in pore radius leads to a higher resistance for pores to escape from the coating. Laser cladding has a high cooling rate, resulting in a large number of pores remaining in the coating and an increase in pore area. Therefore, a proper increase in ultrasonic vibration power and frequency can reduce coating defects, while an excessive increment will generate more coating defects [23].

### 3.3. Influences of Ultrasonic Vibrations on Hardness

**Figure 11:**
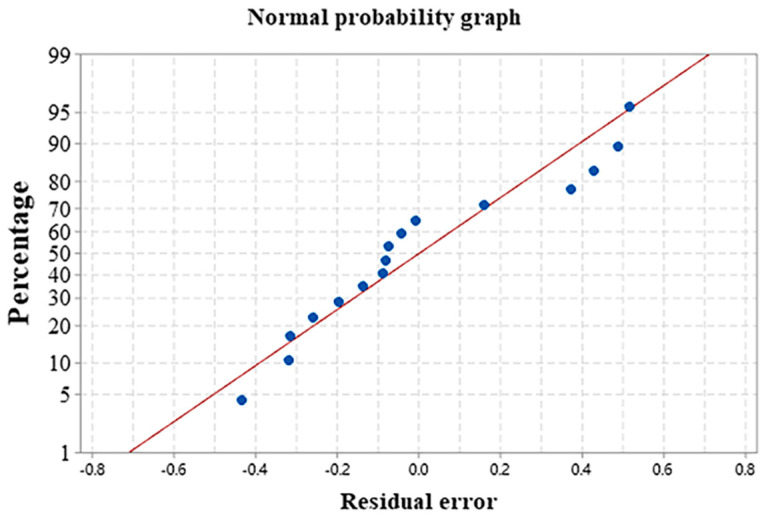
Normal hardness distribution.

The fitting regression model is adopted in this research. Figure 11 presents the normal distribution of the experimental results. The residual value is fitted around the prediction straight line with an S-shape distribution, suggesting the normal distribution of the data and the reliability of the model [24]. Equation (5) provides the hardness mathematical model obtained by regression analysis. Table 4 lists the results of the variance analysis.

(5) Hardness=19.63+0.0561UP+1.182F−0.000023UP×UP−0.01701F×F−0.000930UP×F

The *p*-value is less than 0.01, indicating that the model has a high precision (see Table 6). R^2^, R^2^_adj_, and R^2^_pre_ are more approximate to 100%, suggesting higher fitting accuracy of the model with smaller errors. Less than a 20% difference between R^2^_adj_, and R^2^_pre_ implies remarkable fitting accuracy of the model. Table 6 demonstrates that UP, F, and the interaction items of UP and F significantly affect the model.

**Figure 12:**
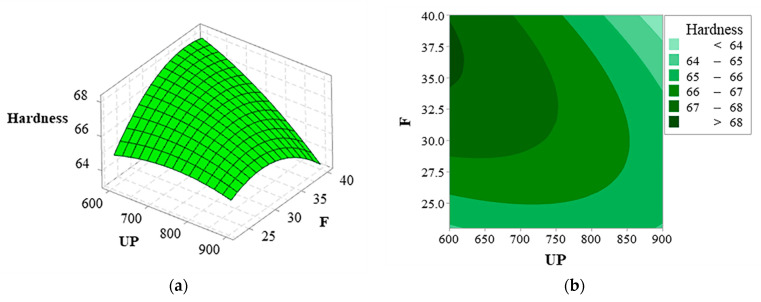
Interaction diagram of UP and F: (**a**) surface diagram and (**b**) contour diagram.

Figure 12a,b provide the surface and contour plots of coating hardness affected by ultrasonic power and frequencies. Hardness increases and then decreases with increased ultrasonic power and frequencies.

**Figure 13:**
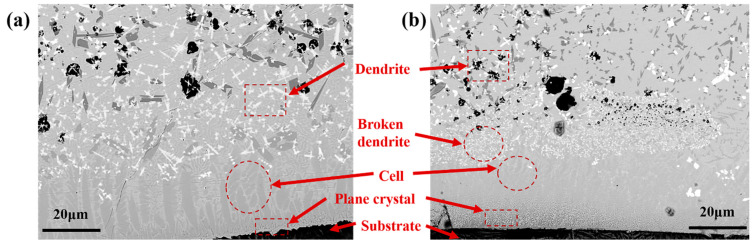
Microstructures of the junction between the cladding layer bottom and the substrate (**a**) without ultrasonic vibrations and (**b**) with ultrasonic vibrations (UP = 800 W, F = 32 kHz).

Figure 13a,b present the microstructure of the junction between the cladding layer bottom and the substrate with and without ultrasonic vibrations. Figure 13a indicates that the grains at the bottom of the coating and the substrate are mainly planar and cellular crystals. As the temperature gradient decreases, most grains are transformed into dendrite crystals with unmelted B_4_C particles remaining in the coating. Based on Figure 13b, the cellular crystals in the coatings significantly reduce in size with a finer distribution under ultrasonic vibrations. The cavitation bubble generated by ultrasonic vibration will form a high-speed shock wave at the moment of collapse. The bottom dendrites are ruptured by the instantaneous spatiotemporal bubbles to produce the impact force to destroy the primary dendrites and form more nucleation points. Further, bubbles created by the cavitation effect absorb a large amount of heat from the surrounding molten pool in the expansion process, increasing the undercooling of local areas. A large surface tension gradient strengthens solution flow in the molten pool, causing the dendrites to break. The dendrite fragments act as the nuclei of new grains [27,28,29] to grow more fine grains.

The acoustic flow effect generated by ultrasonic vibrations promotes thermal convection and accelerates the cooling of the molten pool, causing a decrease in grain size [30]. According to the Hall–Petch relationship, the finer grains create a larger total area of grain boundaries. More dislocations are accumulated at the grain boundaries during deformation to produce higher dislocation resistance, thus increasing the coating hardness. Equation (6) shows the grain-boundary strengthening mechanism (Δ*σ_gb_*) [31].

(6) $$\Delta \sigma_{gb}=kd^{-1/2}$$

where *k* is the material constant; *d* is the average particle size. The grain-boundary strengthening ability negatively correlates with particle sizes. Grain refinement enhances the coating strength and hardness, as fine grains have large boundary areas to hinder grain dislocation.

**Figure 14:**
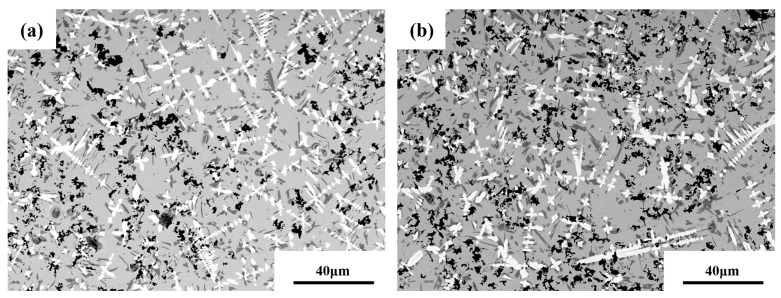
Microstructure at the coating top (**a**) with and (**b**) without ultrasonic vibrations (UP = 800 W, F = 32 kHz).

According to Figure 13, the number and size of black unmelted B_4_C particles significantly decrease due to the ultrasonic cavitation effect that can accelerate the melting of unmelted particles [18]. Unmelted B_4_C particles decrease, followed by morphology from irregular polygons to spherical, eliminating the agglomeration of nano B_4_C powders in the molten pool to a certain extent. Due to the diffusion attenuation of ultrasonic waves, this research applies ultrasonic waves at the substrate bottom. As ultrasonic waves propagate in the molten pool, resistance in the pool causes energy loss during ultrasonic wave propagation. Therefore, the grain morphology does not change evidently at the coating top [32,33] (see Figure 14).

Excessive ultrasonic vibrations create the cavitation effect to produce more energy for coatings, slowing the cooling rate of the molten pool for sufficient grain growth in the molten pool. The strengthening of fine grains disappears under the Ostwald Ripening effect [34]. Coarse grains develop to reduce coating hardness. Therefore, the cavitation effect refines grains to increase hardness. However, when the cavitation effect reaches saturation, high temperatures and high pressures are generated to grow grains. The coarse dendrites reduce the total surface area of the grain and subgrain boundaries, weakening the ability of the coating microstructure to resist dislocations and decreasing the coating hardness.

### 3.4. Influences of Ultrasonic Vibrations on Wear Resistance

**Figure 15:**
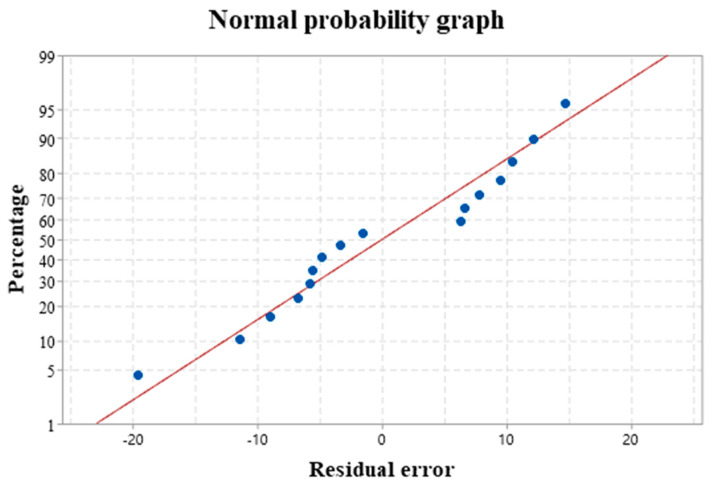
Normal distribution of wear volume.

Figure 15 is the normal distribution of wear resistance analysis. The data conform to the normal distribution as the residual value is fitted around the prediction straight line with an S shape. The reliability of the model is established. Equation (7) provides the mathematical model of wear volume by regression analysis, and Table 6 lists the results of the variance analysis.

(7) $$WearLoss=2150-4.392UP-31.76F+0.002813UP^{2}+0.2802F^{2}+0.02218UP\times F$$

Based on Table 7, the *p*-value of less than 0.01 indicates a high model precision. R^2^, R^2^_adj_, and R^2^_pre_ are closer to 100%, suggesting that the model has higher fitting accuracy with smaller errors. R^2^_adj_, and R^2^_pre_ have less than a 20% difference, implying the remarkable fitting accuracy of the model. Table 7 shows that the model is influenced significantly by UP, F, and the interaction items of UP and F.

**Figure 16:**
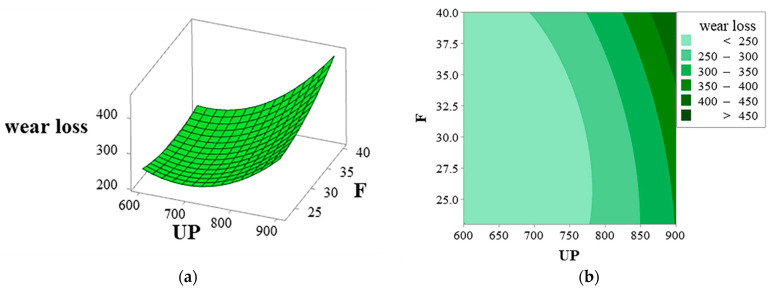
Interaction diagram of UP and F: (**a**) surface diagram and (**b**) contour diagram.

Figure 16a,b provide the surface and contour plots of the interaction between the coating wear volume and ultrasonic power and frequency. The wear volume increases with increasing ultrasonic power and frequencies.

**Figure 17:**
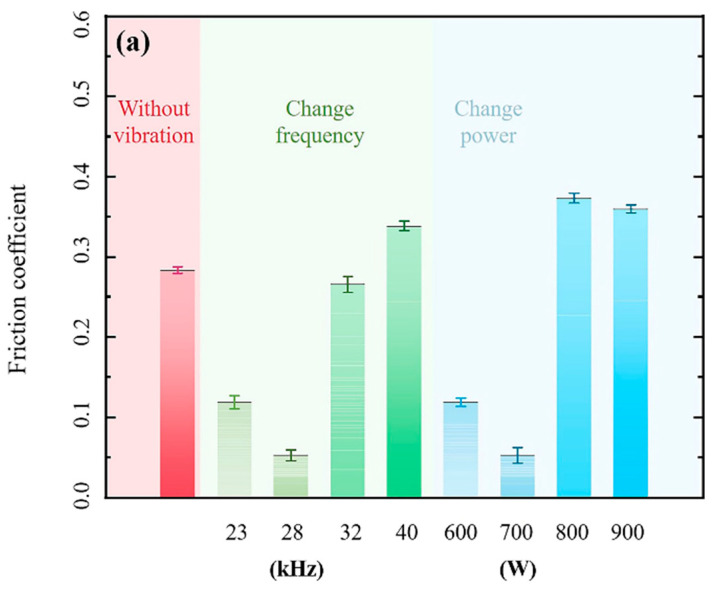

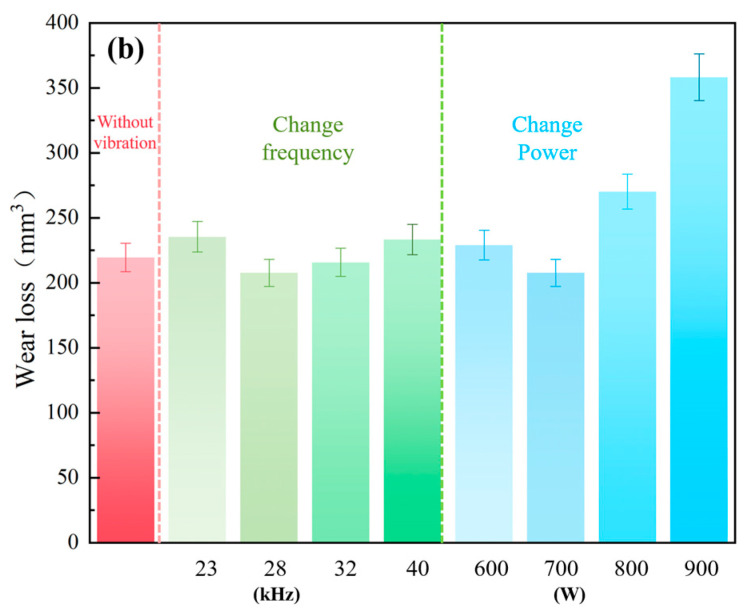
Effects of ultrasonic vibrations on the friction properties of coatings: (**a**) COF and (**b**) wear loss.

Figure 16 explores the impact of the interaction between ultrasonic power and ultrasonic frequency on the coating wear resistance. However, when ultrasonic power is less than 800 W, the interaction does not affect the wear resistance. To better understand the effects of process parameters, a single-factor method is adopted to explore the individual influence of ultrasonic power and frequency on wear resistance. As shown in Figure 17a, COF (Coefficient of friction) value is an important index to evaluate the wear resistance of the cladding layer. The increase in COF diminishes the wear resistance of the cladding layer. The red, green, and blue areas in the figure indicate the COF values without ultrasonic vibration, at 700 W ultrasonic power with varying frequencies, and at 28 kHz frequencies with varying ultrasonic power, respectively. The COF decreases and then increases as ultrasonic frequencies and powers increase. Figure 17b provides the wear volume of coatings, similar to the COF value. The optimal wear resistance of coatings is obtained either at 28 kHz frequencies or at 700 W ultrasonic power. The cavitation effect of ultrasonic vibration refines grains to enhance the coating strength and improve the wear resistance of the coatings. So, the wear volume is minimum.

The cavitation effect of ultrasonic vibration plays the role of grain refinement, which improves the strength of the cladding layer, while the fine and dense grains resist the pressure of the friction pair and increase the coating wear resistance.

**Figure 18:**
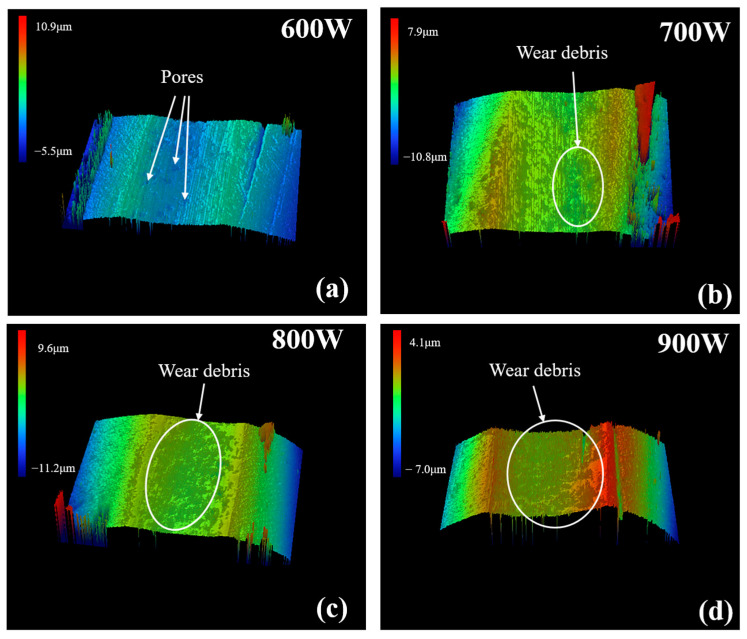
The 3D wear morphologies of coatings under different ultrasonic power: (**a**) 600 W; (**b**) 700 W; (**c**) 800 W; (**d**) 900 W.

Figure 18 shows 3D wear morphologies of coatings at ultrasonic powers of 600, 700, 800, and 900 W, respectively. Figure 18a indicates that the coating has a small wear volume but with pores. The coating in Figure 18b has minimum wear volume and depth. The wear width and depth increase significantly with further increased ultrasonic power, as shown in Figure 18c,d. The maximum wear width and depth are provided in Figure 18d.

## 4. Conclusions

This research synthesized in situ the NbC-reinforced phase on Ni substrates by ultrasonic vibration-assisted laser cladding. This study discusses the influence mechanism of ultrasonic power and frequency on the behavior of a laser cladding molten pool given varying process parameters to explore the change in coating structure and properties. The main conclusions of this paper are as follows:

(1) The coatings mainly consist of FeNi_3_, NbC, B_4_C, and CrB_2_ phases. The application of ultrasonic vibration does not change the phase composition of the coating, but significantly reduces the grain size of the coating to achieve a finer and more uniform distribution. The cavitation bubbles generated by ultrasonic vibration can create high-speed shock waves at the collapse moment. The shock waves destroy the primary dendrite to form more nucleation points. So, the grain size of the coating is decreased.
(2) Ultrasonic vibration effectively reduces the pore area of the coating. With the increase in ultrasonic frequency and power, the pore area of the cladding layer decreases first and then increases. The coating has the smallest pore area when ultrasonic power is 800 W and ultrasonic frequency is 32 kHz. The collapse of cavitation bubbles generated by ultrasonic vibration can produce instantaneous high temperature and high pressure in the surrounding local areas. Consequently, local excitation waves are created around the bubbles. The surrounding bubbles are then crushed, thus reducing the coating pore area.
(3) Ultrasonic vibration effectively improves the coating hardness. As ultrasonic power and frequency increase, the coating hardness first increases and then decreases. The coating hardness is maximized at 600 W ultrasonic power and 40 kHz ultrasonic frequency. The cavitation effect destroys the coarse dendrites and increases the hardness of the coating.
(4) Ultrasonic vibration enhances the wear resistance of the coating. The wear resistance increases and then decreases when ultrasonic power and frequency increase. The wear resistance is the highest when ultrasonic power is 700 W and ultrasonic frequency is 32 kHz. There is a positive relationship between wear resistance and hardness. As the coating hardness is high, the fine and dense grains resist the pressure of the friction pair, thus improving the wear resistance.

## Figures and Tables

**Table 1:** Chemical composition of Ni45 (wt%).

	C	Cr	Si	Fe	B	O	Ni
Ni45A	0.32	7.75	3.35	2.75	1.65	<0.05	Balance

**Table 2:** Experimental factors and levels.

Process Parameters	Mark	Units	Levels			
			1	2	3	4
Ultrasonic power	UP	W	600	700	800	900
Frequency	F	kHz	23	28	32	40

**Table 3:** Test factors and results.

	UP (W)	F (kHz)	Pore Area (μm^2^)	Hardness (HRC)	Wear Loss (mm^3^)
1	600	23	37,140	65.283	245.546
2	600	28	25,231	66.714	230.509
3	600	32	23,748	67.400	223.971
4	600	40	22,898	67.950	237.798
5	700	23	28,734	65.250	228.740
6	700	28	18,829	66.763	219.049
7	700	32	13,550	66.933	221.383
8	700	40	23,591	67.350	252.954
9	800	23	24,069	65.043	262.447
10	800	28	14,096	66.525	263.845
11	800	32	13,700	66.929	275.052
12	800	40	19,719	65.163	324.368
13	900	23	21,144	64.729	352.410
14	900	28	20,303	65.980	364.899
15	900	32	16,109	65.200	384.978
16	900	40	27,655	63.025	452.039

**Table 4:** Point scan results marked at different positions in Figure 4a.

Marked Locations	B	C	Cr	Fe	Nb	Ni
A	0.77	36.85	8.09	0.00	52.03	2.26
B	10.99	16.61	40.92	18.75	4.38	8.35
C	7.14	54.06	5.68	1.15	0.00	31.97

**Table 5:** Variance analysis of the pore area.

Source	DF	Adj SS	Adj MS	F-Value	*p*-Value
Regression	5	543,164,601	108,632,920	26.69	<0.001
UP	1	151,522,861	151,522,861	37.23	<0.001
F	1	350,210,743	350,210,743	86.05	<0.001
UP × UP	1	89,965,225	89,965,225	22.11	<0.001
F × F	1	248,221,020	248,221,020	60.99	<0.001
UP × F	1	90,575,980	90,575,980	22.26	<0.001
Error	10	40,696,794	4,069,679		
Total	15	583,861,395			
R^2^ = 93.03%		R^2^_adj_ = 89.54%		R^2^_pre_ = 80.46%	

**Table 6:** Variance analysis of the hardness.

Source	DF	Adj SS	Adj MS	F-Value	*p*-Value
Regression	5	23.0849	4.6170	33.02	<0.001
UP	1	2.0504	2.0504	14.66	0.003
F	1	10.8130	10.8130	77.33	<0.001
UP × UP	1	0.8434	0.8434	6.03	0.034
F × F	1	5.0828	5.0828	36.35	<0.001
UP × F	1	6.6947	6.6947	47.87	<0.001
Error	10	1.3984	0.1398		
Total	15	24.4833			
R^2^ = 94.29%		R^2^_adj_ = 91.43%		R^2^_pre_ = 82.84%	

**Table 7:** Variance analysis of wear volume.

Source	DF	Adj SS	Adj MS	F-Value	*p*-Value
Regression	5	74,065	14,812.9	101.80	<0.001
UP	1	12,581	12,581.3	86.47	<0.001
F	1	3320	3320.1	22.82	<0.001
UP × UP	1	12,659	12,659.4	87.00	<0.001
F × F	1	1379	1379.4	9.48	0.012
UP × F	1	3807	3806.7	26.16	<0.001
Error	10	1455	145.5		
Total	15	75,520			
R^2^ = 98.07%		R^2^_adj_ = 97.11%		R^2^_pre_ = 94.47%	
